# Clinical characteristics of pediatric pertussis cases, Quebec 2015–2017

**DOI:** 10.14745/ccdr.v44i09a01

**Published:** 2018-09-06

**Authors:** M Desjardins, D Iachimov, S Mousseau, P Doyon-Plourde, N Brousseau, F Rallu, C Quach

**Affiliations:** 1 Medical Microbiology and Infectious Diseases, Centre Hospitalier de l’Université de Montréal, Montréal, QC; 2 Research Institute, Centre Hospitalier Universitaire Sainte-Justine, Montréal, QC; 3 Pediatric Emergency Division, Department of Pediatrics, Centre Hospitalier Universitaire Sainte-Justine, Montréal, QC; 4 Department of Microbiology, Infectious Diseases, and Immunology, Faculty of Medicine, University of Montreal, Montréal, QC; 5 Biological Risks and Occupational Health Division, Institut national de santé publique du Québec, Quebec City, QC; 6 Department of Medical Microbiology, Centre Hospitalier Universitaire Sainte-Justine, MontréalQC; 7 Infection Prevention & Control, Centre Hospitalier Universitaire Sainte-Justine, Montréal, QC; 8 Chair, National Advisory Committee on Immunization

**Keywords:** pertussis, pediatric, symptoms, outcomes, Quebec

## Abstract

**Background:**

The introduction of the acellular pertussis vaccine may have changed the epidemiological and clinical features of pertussis in Canadian children.

**Objective:**

To describe the demographics, clinical presentation and outcomes of children and adolescents with pertussis presenting to a tertiary care hospital.

**Methods:**

Retrospective cohort of consecutive patients evaluated at the Centre Hospitalier Universitaire Sainte-Justine (CHUSJ) and tested with a bacterial multiplex real-time polymerase chain reaction (PCR) for *Bordetella pertussis* or *B. parapertussis* between June 2015 and March 2017. Demographics, clinical presentations and outcomes were described for positive test results. The Modified Preziosi Scale was used to assess disease severity; severe disease was defined as a score ≥7.

**Results:**

The age distribution of the 144 positive patients with a clinical encounter at CHUSJ was as follows: less than three months (n=25/144, 17.4%), four months to nine years (n=63/144, 43.8%) and 10 to 18 years (n=56/144, 38.9%). The most common symptoms at presentation were paroxysmal cough (70.1%), post-tussive emesis (47.2%) and coryza (33.3%). Over 84.0% of cases in infants less than three months of age had severe pertussis (92.0% required hospitalization and 28.0% intensive care admission). In children four months to nine years of age, 22.2% had severe pertussis and 11.1% required hospitalization. Only two (3.6%) children greater than 10 years had severe disease.

**Conclusion:**

Pertussis still affects children of all ages in Quebec. In older children, it tends to be a milder disease. When it affects infants, who do not yet have full protection from pertussis vaccination, it often causes severe disease, especially in those less than three months of age. This evidence further supports the implementation of a pertussis vaccination program in pregnant women.

## Introduction

Pertussis, or whooping cough, is a respiratory tract infection caused by *Bordetella pertussis* and *B. parapertussis*. Infected patients may display a wide range of symptoms depending on age, immunization status and coinfections, often making pertussis difficult to diagnose (1,2).

Pertussis is a vaccine-preventable disease. Vaccination against pertussis with a whole cell vaccine, which was introduced in Canada in 1943, led to a significant decrease in the disease incidence (3). The whole cell vaccine was replaced with the acellular pertussis vaccine in the late 1990s to decrease the incidence of adverse events following immunization.

The pertussis-containing vaccine is currently administered at two, four, six and 18 months of age with a booster between four and six years of age. In Quebec, the universal acellular vaccination program was introduced in 1998 with a marked impact on pertussis incidence (4-6). In 2016, despite vaccination coverage of 97.3% in children at one year of age (7), the incidence of pertussis in those less than 18 years of age was still 60 cases per 100,000 children (8).

It has been shown that immunity and protection provided by the acellular vaccine wanes rapidly (9), and this may have changed the clinical presentation of pertussis in children. Young infants are particularly vulnerable to pertussis, possibly because those less than three months of age have only received one dose of pertussis vaccine, which provides only partial protection (6,10,11). To address this, the National Advisory Committee on Immunization (NACI) evaluated the evidence on vaccination of pregnant women and found this to be highly effective in preventing pertussis in infants (12-14). In 2018, NACI recommended the immunization of pregnant women against pertussis (15), noting it could lead to a 90% reduction of the incidence of pertussis in infants born to vaccinated mothers (16).

The last hospital-based studies describing the epidemiological and clinical features of pertussis in Canadian children were conducted from 1991 to 2004 (17,18); thus, the current burden of illness in children in Canada is currently unknown.

The objective of this study was to describe the clinical presentation and outcomes of children with pertussis who were evaluated between June 2015 and March 2017 at the Centre Hospitalier Universitaire Sainte-Justine (CHUSJ). This hospital is in Montreal (Quebec) and is the only free-standing children’s hospital in the province of Quebec, with 80,000 emergency care visits annually (19).

## Methods

### Study design

This was a retrospective, observational cohort study of consecutive patients evaluated at CHUSJ for suspected pertussis. Children presenting for suspected pertussis were primarily from the hospital’s catchment area and were assessed in the emergency department. Occasionally, children were tested for pertussis as inpatients. All children were tested with a bacterial multiplex polymerase chain reaction (PCR) (*B. pertussis*, *B. parapertussis*, *B. holmesii*, *Mycoplasma pneumoniae* and *Chlamydophila pneumoniae*). Since 2015, all suspected pertussis cases seen at CHUSJ are tested using this multiplex PCR.

Cases positive for *B. pertussis* or *B. parapertussis* between June 2015 and March 2017 were identified through the laboratory information system and clinical data were extracted using manual chart review. The study protocol was approved by the CHUSJ ethics committee.

### Study population

The study included consecutive patients aged zero to 17 years, who had a positive multiplex PCR (cycle threshold [Ct] value less than 36) for *B. pertussis* or *B. parapertussis,* and whose clinical and laboratory data were available in the CHUSJ microbiology laboratory information system between June 2015 and March 2017. Since *B. parapertussis* may cause a disease similar to pertussis and the current vaccine against *B. pertussis* may offer cross-protection to *B. parapertussis* (20), patients with *B. parapertussis-*positive PCR were included in the study. Patients with equivocal PCR results (Ct values 36–39.9) were also included, as they are currently considered as pertussis cases by public health authorities in Quebec, if they present symptoms compatible with pertussis (6). Patients who tested positive for *B. holmesii* were not included, since this *Bordetella* species may cause a significant different disease (1). Patients 18 years and older, as well as patients without a clinical encounter at CHUSJ (e.g., samples received from other hospitals), were excluded.

### Data collection and analysis

In addition to reviewing the laboratory data, manual chart reviews of electronic medical records were performed (using Chartmaxx; Quest Diagnostics, Secaucus, New Jersey, United States [US]), using standardized case report forms to collect information on 1) clinical presentation (using triage nurses’ evaluation and physicians’ clinical notes on the day the PCR was ordered), 2) investigation results and 3) outcomes (hospitalization, length of stay, macrolide treatment, intensive care admission or death). Data collection was performed by two members of the research team (MD, DI) and 10% of the charts were reviewed by both researchers to evaluate inter-rater agreement (tested using kappa statistics). Patients were divided in three age groups, as suggested at the Global Pertussis Initiative roundtable meeting held in February 2011 (21): less than or equal to three months; four months to nine years; and 10 to 18 years of age. Absolute numbers and proportions were used to analyze demographics, clinical presentations and outcomes. Interquartile range (IQR) was used to evaluate the statistical dispersion of continuous variables. The Modified Preziosi Scale (MPS) (22) was used to assess disease severity. Severe pertussis was defined by MPS score greater or equal to seven (23,24). Microsoft Excel 2016 (Redmond, Washington, US) was used to generate proportions and IQR. Statistical analyses were descriptive.

## Results

Of the 1,526 multiplex PCR tests performed between June 11, 2015 and March 31, 2017, 173 patients were positive or equivocal for *B. pertussis* or *B. parapertussis* (11.3% positivity). Twenty-nine patients were excluded: two were 18 years or older and 27 did not have a clinical encounter at CHUSJ.

### Demographics

A total of 144 patients were analyzed: 133 *B. pertussis* cases (109 positive, 24 equivocal); and 11 *B. parapertussis* cases (seven positive, four equivocal) (**Table 1**). Patients were pooled together for analysis because of the small number of patients who tested positive for *B. parapertussis* and because both bacteria cause similar respiratory syndromes.

**Table 1:** Characteristics of children with PCR-confirmed pertussis

**Characteristics**	**Age groups,** n (%)			
	**0–3 mos**	**4 mos – 9 yrs**	**10–18 yrs**	**Total**
**Laboratory result**	25 (17.4)	63 (43.8)	56 (38.9)	144 (100.0)
*B. pertussis* positive	19 (76.0)	45 (71.4)	45 (80.4)	109 (75.7)
*B. parapertussis* positive	1 (4.0)	5 (7.9)	1 (1.8)	7 (4.9)
*B. pertussis* equivocal	5 (20.0)	9 (14.3)	10 (17.9)	24 (16.7)
*B. parapertussis* equivocal	0 (0.0)	4 (6.3)	0 (0.0)	4 (2.8)
**Female**	14 (56.0)	33 (52.4)	35 (62.5)	82 (56.9)
**Past medical history**				
Asthma	0 (0.0)	11 (17.5)	11 (19.6)	22 (15.3)
Immunization status up to date	22 (88.0)	42 (66.7)	48 (85.7)	112 (77.8)
Prematurity	4 (16.0)	4 (6.3)	1 (1.8)	9 (6.3)

Abbreviations: *B.*, *Bordetella*; mos, months; PCR, polymerase chain reaction; yrs, years

**Figure 1:**
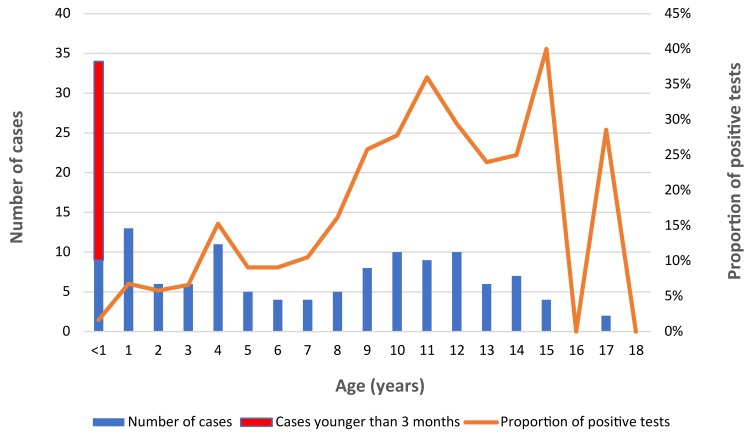
Distribution of patients with positive pertussis PCR per age (n=144) Abbreviations: n, number; PCR, polymerase chain reaction

Among the 144 children, 25 (17.4%) were less than three months old, 63 (43.8%) were between four months and nine years and 56 (38.9%) were between 10 and 18 years. The proportion of positive tests increased with age, reaching a peak of 35–45% in adolescents 10 to 15 years old (**Figure 1**).

**Figure 2:**
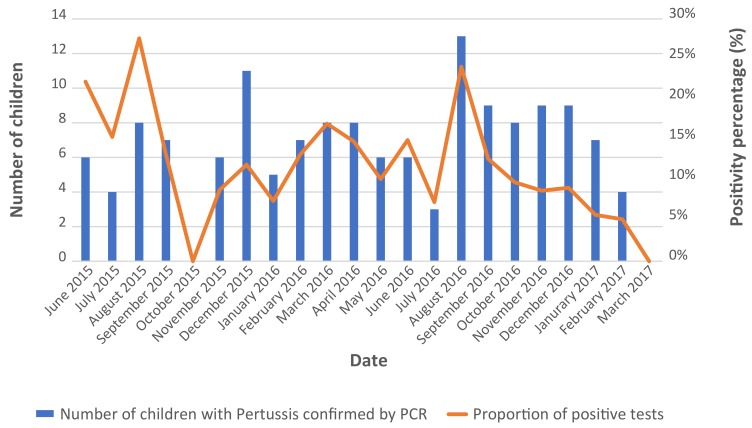
Distribution of patients with positive pertussis PCR during the study period (n=144) Abbreviations: n, number; PCR, polymerase chain reaction

Pertussis was reported all year round (**Figure 2**).

### Clinical presentation

The most common symptoms at presentation were paroxysmal cough (70.1%), post-tussive emesis (47.2%) and coryza (33.3%) (**Table 2**). From the 100 chest X-rays performed (69.4% of all cases), only eight (8.0%) were consistent with pneumonia. The 25 children under the age of three months were the most severely affected by pertussis, with a median MPS score of 12 (interquartile range [IQR]: 9-15). The disease was considered severe in 84.0% of children less than three months of age. All 10 reported cases of apnea, 75.0% (n=15/20) of cases with cyanosis, 76.9% (n=10/13) of cases with chest retractions and 45% (n=9/20) of cases with inspiratory whoop were in this age group. Children four months to nine years of age were also significantly affected: 22.2% had severe pertussis. In comparison, only two (3.6%) children older than 10 years of age had severe pertussis. Inter-rater agreement using kappa statistics was 0.86, showing good validity of data collection.

**Table 2:** Clinical presentation and paraclinical tests of children with PCR-confirmed pertussis

**Characteristics**	**Age groups,** n (%)^a^			
	**0–3 mos** n=25	**4 mos – 9 yrs** n=63	**10–18 yrs** n=56	**Total** n=144
**Clinical presentation**				
Paroxysmal cough	20 (80.0)	47 (74.6)	34 (60.7)	101 (70.1)
Inspiratory whoop	9 (36.0)	7 (11.1)	4 (7.1)	20 (13.9)
Post-tussive emesis	10 (40.0)	32 (50.8)	26 (46.4)	68 (47.2)
Cyanosis	15 (60.0)	4 (6.3)	1 (1.8)	20 (13.9)
Chest retractions	10 (40.0)	3 (4.8)	0 (0.0)	13 (9.0)
Fever	2 (8.0)	10 (15.9)	4 (7.1)	16 (11.1)
Coryza	14 (56.0)	20 (31.8)	14 (25.0)	48 (33.3)
Pulmonary signs on exam	8 (32.0)	8 (12.7)	2 (3.6)	18 (12.5)
Apnea	10 (40.0)	0 (0.0)	0 (0.0)	10 (6.9)
Otitis	2 (8.0)	6 (9.5)	2 (3.6)	10 (6.9)
Pharyngitis	2 (8.0)	2 (3.2)	7 (12.5)	11 (7.6)
Sub-conjunctival hemorrhage	0 (0.0)	1 (1.6)	0 (0.0)	1 (0.7)
Seizures	0 (0.0)	0 (0.0)	0 (0.0)	0 (0.0)
**MPS**				
Average	11.8	4.7	3.3	5.4
Median	12	5	3	5
− IQR 25–75	9–15	3–6	2–5	3–7
Severe cases (MPS ≥7)	21 (84.0)	12 (22.2)	2 (3.6)	37 (25.7)
**Paraclinical tests**				
Viral multiplex done	13 (52.0)	6 (9.5)	5 (8.9)	24 (16.7)
− Respiratory virus found^b^	5 (38.5)	2 (33.3)	1 (20.0)	8 (33.3)
Complete blood count done	21 (84.0)	10 (15.9)	3 (5.4)	34 (23.6)
− Lymphocytosis^b^	10 (47.7)	3 (30.0)	0 (0.0)	13 (38.2)
Chest x-ray done	20 (80.0)	43 (68.3)	37 (66.1)	100 (69.4)
− Pneumonia identified^b^	0 (0.0)	6 (14.0)	2 (5.4)	8 (8.0)

Abbreviations: IQR, interquartile range; mos, months; MPS, Modified Preziosi Scale; n, number; yrs, years

^a^ All results reported as n (%) with the exception of MPS average, MPS median (and IQR)

^b^ These percentages were calculated according to the number of respective tests done

### Outcomes

Overall, 20.8% of patients were hospitalized (**Table 3**). Infants less than three months of age had the highest risk of hospitalization (92%) with a significant proportion (28%) requiring intensive care admission. In comparison, 11% of children four months to nine years of age and none of older children were hospitalized. The majority of patients (75.2%) were treated with macrolides. There were no deaths.

**Table 3:** Outcomes of children with PCR-confirmed pertussis

**Outcomes**	**Age groups,** n (%)^a^			
	**0–3 mos** n=25	**4 mos – 9 yrs** n=63	**10–18 yrs** n=56	**Total** n=144
Hospitalization	23 (92.0)	7 (11.1)	0 (0.0)	30 (20.8)
**Length of stay**				
Average, days	11.0	3.0	0.0	9.1
Median	8	3	0	5
− IQR 25–75	3–14	2–4	0	3–12
Intensive care	7 (28.0)	0 (0.0)	0 (0.0)	7 (4.9)
Death	0 (0.0)	0 (0.0)	0 (0.0)	0 (0.0)
Received macrolide	18 (72.0)	46 (73.0)	45 (80.4)	109 (75.7)

Abbreviations: IQR, interquartile range; mos, months; n, number; yrs, years

^a^ All results reported as n (%) with the exception of Length of stay which is average (in days) and Median with IQR

## Discussion

This study draws a brief portrait of the clinical presentation and outcomes of patients with pertussis presenting to a tertiary care pediatric hospital. Despite the introduction of a universal acellular pertussis immunization program in Quebec, infants less than three months of age are still affected by pertussis. Most suffered a severe disease (84%) and required hospitalization (92%), including in the intensive care unit (28%), for a median length of stay of eight days, similar to previous studies (10,18,21,25–31). Disease was milder in older children, as shown by lower MPS scores and hospitalization rates. Only a minority of children had severe pertussis, with no hospitalization in those 10 years or older, suggesting that older children, like adults, were less severely affected by this infection (25,32).

Overall, symptoms suggestive of pertussis, such as paroxysmal cough, inspiratory whoop and post-tussive emesis, were found in a large proportion of children of all ages, but less frequently than what was previously reported (25,29,31). For example, in previous studies children age nine years or younger, paroxysmal cough, inspiratory whoop and post-tussive emesis were previously reported in 89-93% (cough), 69%–92% (whoop) and 48%–60% (emesis) of children. In contrast, in our study these symptoms were present in 76% (cough), 25% (whoop) and 48% (emesis) of children of similar age. These differences could be due in part to an attenuation of disease following immunization, but could also be the result of the increased sensitivity of PCR compared with culture, which allows for detection of less symptomatic cases (33). It was previously shown that immunity and protection provided by the acellular vaccine wanes rapidly (9), which may explain the high proportion of positive tests in children 10-15 years of age (25%–40%).

This study offers a description of pediatric cases of pertussis in Quebec. There are several limitations to consider. First, this study was from a single centre; nevertheless, the 144 cases analyzed in this study represented approximately 12.4% of all pertussis cases <18 years of age diagnosed in the province of Quebec during the study period (8). Second, there was the risk of information bias, including potential misclassification of the vaccination status, an intrinsic risk associated with chart review. We tried to minimize the risk by documenting a high inter-rater agreement. Third, we analyzed only patients who sought medical attention in a hospital setting, which may overestimate the severity of disease. However, this bias is probably not significant in infants less than three months of age, as reported cases of pertussis in this age group usually seek emergency care and are hospitalized (34). Finally, the study period included the peak of a four-year epidemic cycle (2016), which could have led to a slight overestimation of the incidence of pertussis.

In terms of next steps and future research, this study could provide a baseline for a future evaluation of the impact of the vaccination of pregnant women on the pertussis disease burden in young children.

### Conclusion

Pertussis still affects children of all ages in Quebec. In older children it tends to be a milder disease. When it affects infants, who do not yet have full protection from pertussis vaccination, it often causes severe disease, especially in those less than three months of age. This evidence further supports the implementation of a pertussis vaccination program in pregnant women and provides a baseline to assess the impact of this program.
